# Impact of Simulation-Based Training on Maternal and Neonatal Outcomes in Delivery Rooms: A Systematic Review

**DOI:** 10.7759/cureus.101123

**Published:** 2026-01-08

**Authors:** Louise Raeymaekers, Ludivine Balant, Deborah Goldman, Yoann Marechal

**Affiliations:** 1 Obstetrics and Gynecology, CHU Charleroi-Chimay, Charleroi, BEL; 2 Anesthesiology, CHU Charleroi-Chimay, Charleroi, BEL; 3 Neonatology, CHU Charleroi-Chimay, Charleroi, BEL

**Keywords:** massive postpartum hemorrhage, newborn resuscitation, perinatal simulation-based training, postpartum hemorrhage, shoulder dystocia prevention

## Abstract

This systematic review examines the impact of simulation-based training (SBT) in delivery rooms on maternal and neonatal outcomes in high-resource settings. Given the persistently high rates of maternal and neonatal mortality worldwide, SBT has emerged as a promising method to enhance clinical performance, decision-making, and teamwork in obstetric emergencies. Eight studies published between 2014 and 2023 were included, covering over 177,000 deliveries. The composite analysis showed a significant reduction in adverse outcomes, including postpartum hemorrhage requiring transfusion and neonatal Apgar scores <7 at five minutes, dropping from 6.9% pre-SBT to 3.8% post-SBT. The improvements were more pronounced in neonatal outcomes than maternal ones. Despite these encouraging findings, the review highlights several limitations: lack of standardization in training protocols, heterogeneity in study designs and outcome measures, and limited data on long-term effects. Additionally, generalizing results to low-resource settings remains a challenge. The authors call for future research using individual patient data, standardized training approaches, cost-effectiveness evaluations, and innovative methods to adapt SBT for broader global use, ultimately aiming to strengthen perinatal safety worldwide.

## Introduction and background

Simulation-based training (SBT) has revolutionized medical education, particularly in high-risk fields such as obstetrics, where unpredictable emergencies demand rapid decision-making, technical precision, and effective teamwork [1,2]. By providing a controlled environment for practice, simulation enables healthcare providers to develop and refine these skills without compromising patient safety. As a result, SBT has become an increasingly integral component of obstetric and neonatal training programs.

Globally, maternal and neonatal mortality remain significant public health challenges, with approximately 800,000 maternal deaths and 2.3 million neonatal deaths annually [3,4]. These deaths are often attributable to preventable complications, including postpartum hemorrhage (PPH), pre-eclampsia, and neonatal asphyxia. Simulation training has been proposed as a means of reducing these complications by improving clinical performance, communication, and team-based decision-making in high-pressure situations.

However, the implementation of simulation programs is highly variable, with significant differences in training modalities, program structures, and outcomes across countries and institutions. This lack of standardization reflects an absence of international consensus on simulation training protocols, complicating efforts to evaluate its overall effectiveness. Furthermore, most existing research focuses on short-term outcomes, leaving questions about the long-term impact of simulation, such as skill retention and cost-effectiveness, largely unanswered [5,6].

This study aims to evaluate the impact of SBT on maternal and neonatal outcomes in delivery rooms, focusing on data from high-resource environments. By pooling outcomes from diverse clinical scenarios, including PPH, neonatal asphyxia, and shoulder dystocia, this review seeks to provide a comprehensive assessment of SBT’s effectiveness while highlighting the challenges and opportunities for future research.

Materials and methods

Search Strategy

A systematic search was conducted using PubMed, Cible+, CISMeF, LiSSa, and the Cochrane Library to identify studies published between 2014 and 2023 (search methodology described in Appendices). Studies were included if they evaluated SBT in delivery room settings and reported clinical outcomes related to maternal and neonatal complications. Specifically, eligible studies provided pre- and post-SBT data and were conducted in high-resource healthcare settings.

The inclusion criteria also required that studies report outcomes such as PPH (defined as requiring at least one packed red cell transfusion), neonatal Apgar scores, or shoulder dystocia-related trauma, ensuring their relevance to obstetric emergencies. Studies were excluded if they lacked clinical outcome data, focused exclusively on low-resource settings, or presented results in formats unsuitable for statistical analysis, such as aggregated or incomplete data that could not be included in pooled analyses. These criteria ensured the inclusion of methodologically robust studies with comparable data to evaluate the effectiveness of SBT.

This rigorous selection process ensured that the studies included in the pooled analysis were methodologically robust and provided comparable data on key maternal and neonatal outcomes.

Composite Analysis

To evaluate the global impact of SBT, a composite analysis was performed, pooling data from three key outcomes: hemorrhages requiring transfusion of ≥1 packed red cell, neonatal Apgar scores <7 at five minutes, and shoulder dystocia-related neonatal injuries.

Maternal outcomes (e.g., PPH) were analyzed at the level of deliveries, while neonatal outcomes (e.g., Apgar scores) were analyzed at the level of newborns. In cases of multiple births (e.g., twins), neonatal outcomes were treated independently, whereas maternal outcomes were counted once per delivery. As maternal and neonatal data are derived from the same deliveries, they are not entirely independent, and this overlap may result in some double-counting when pooling data. This approach was chosen to provide a comprehensive assessment of SBT impact across both maternal and neonatal populations, but the potential for overestimation of event counts is acknowledged in the discussion.

Statistical Analysis

While meta-analysis is a powerful tool for synthesizing data across studies, its applicability in this context is limited due to the heterogeneity of outcomes and study designs. The current systematic review includes studies with varying intervention modalities, outcomes (e.g., transfusion rates vs. Apgar scores), and methodologies, which complicates the pooling of effect sizes. Moreover, the relatively small number of included studies (n=8) and the absence of individual patient data (IPD) restrict the use of advanced meta-analytic techniques such as subgroup analysis or meta-regression. Future research incorporating standardized outcome measures and IPD could enable the application of robust meta-analysis to better quantify the global impact of SBT [5,7].

Quantitative data were therefore analyzed using chi-square tests, with results considered statistically significant for p<0.05. Risk ratios (RRs) and the number needed to treat (NNT) were calculated to quantify the relative and absolute risk reductions using GraphPad Prism 10.4.0 (Dotmatics, Boston, MA, US).

## Review

Results

**Figure 1:**
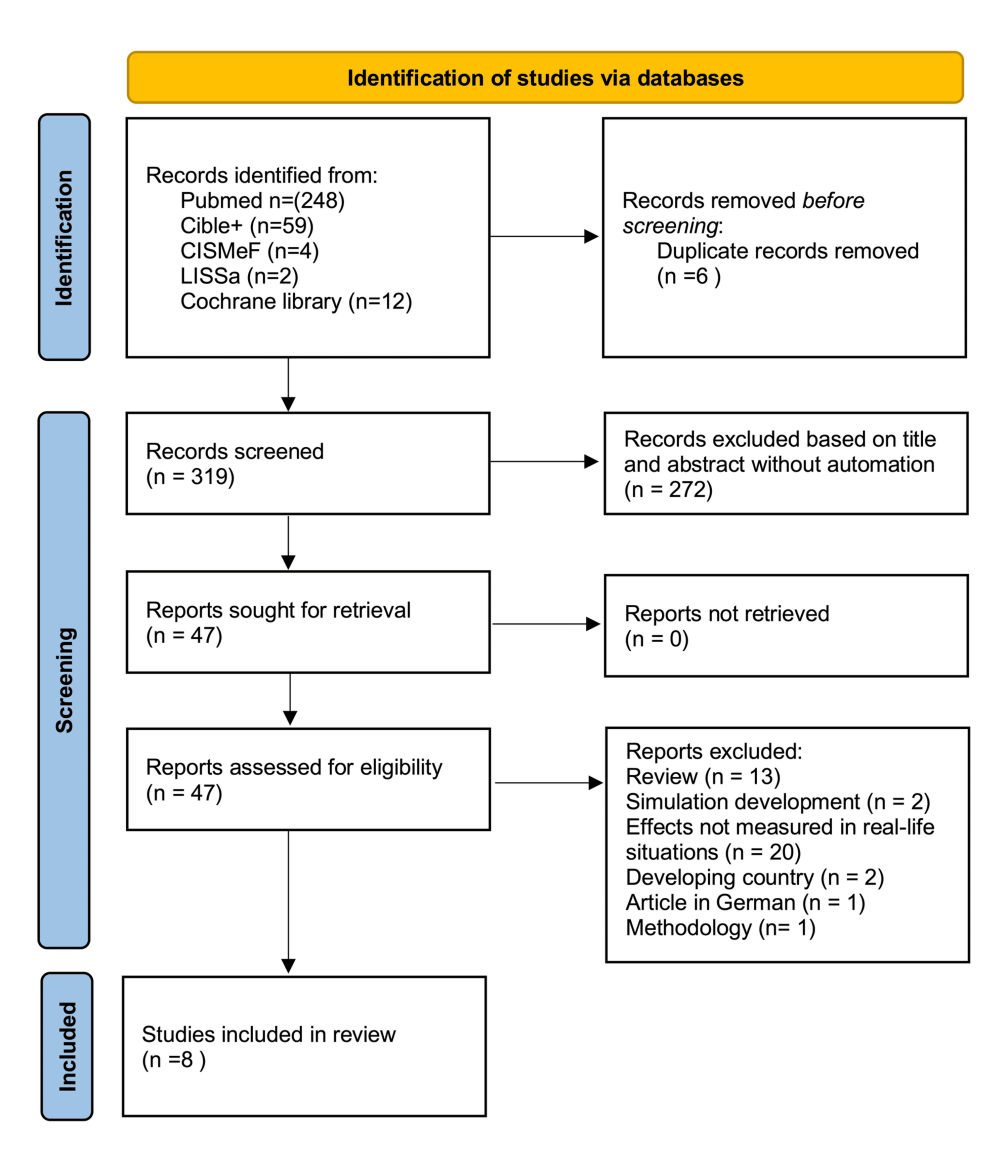
PRISMA flow diagram PRISMA, Preferred Reporting Items for Systematic Reviews and Meta-Analyses

As depicted in the Preferred Reporting Items for Systematic Reviews and Meta-Analyses (PRISMA) diagram, the search yielded 325 potentially relevant studies. After screening titles and abstracts, 47 studies were assessed in full text for eligibility (Figure 1). Finally, eight studies met the inclusion criteria, representing a total of 177,259 deliveries and generating 143,413 statistically analyzed points (92,313 pre-SBT and 51,100 post-SBT) based on the composite score.

Study Characteristics

The studies of interest are heterogeneous in terms of design, clinical focus, and simulation modalities, as summarized in Table 1 [8-15]. The training modalities ranged from hybrid simulations using low-cost tools to high-fidelity mannequins, with study designs including prospective, retrospective, and multicenter approaches. The data selection process ensured the inclusion of studies with rigorous methodologies and relevant outcome measures, contributing to the robustness of the pooled analysis. Maternal outcome, PPH, was recorded per delivery, while neonatal outcomes, such as Apgar scores <7 and trauma related to shoulder dystocia, were recorded per newborn. This dual focus allowed for a comprehensive evaluation of SBT’s impact but introduced some overlap between maternal and neonatal data points when multiple newborns were involved in a single delivery.

**Table 1:** Characteristics of included studies PPH, postpartum hemorrhage

Reference	Study design	Subject	Number of patients	Training modality	Outcome assessed
Egenberg et al., 2016 [8]	Pre-post intervention	PPH management	5,446	Hybrid simulation (MamaNatalie)	Maternal transfusion rates
Dillon et al., 2021 [9]	Observational prospective	PPH management	24,719	Hybrid simulation (MamaNatalie)	Uterotonic use, transfusion response times
Lutgendorf et al., 2023 [10]	Multicenter pre-post	PPH management	9,980	Hybrid simulation (MamaNatalie)	Transfusion rates, uterotonic use
Xu et al., 2023 [11]	Prospective intervention	Neonatal asphyxia management	29,759	Mannequin-based simulation (SimNewB)	Neonatal Apgar scores, asphyxia
Chitkara et al., 2023 [12]	Prospective observational	Preterm infant management	17,283	Hybrid simulation with preterm neonate mannequins	Neonatal mortality, adaptation
Dahlberg et al., 2018 [13]	Retrospective quantitative	Shoulder dystocia	33,853	Human and mannequin simulation	Neonatal injury rates
Fransen et al., 2016 [14]	Randomized controlled multicenter	Obstetric emergencies	56,166	High-fidelity mannequins (Noelle™, ECS™)	Maternal and neonatal outcomes
Olson et al., 2021 [15]	Retrospective cohort	Shoulder dystocia management	53	Human and mannequin simulation	Standardization of maneuvers, neonatal outcomes

The primary outcome of this review was the composite reduction in adverse maternal and neonatal events, which included hemorrhages requiring transfusion, massive maternal transfusion, and neonatal Apgar scores <7 at five minutes (Table 2). The composite analysis demonstrated a significant reduction in adverse outcomes from 6.9% pre-SBT to 3.8% post-SBT, corresponding to a 45% relative risk reduction (RR=0.55) and an absolute risk reduction of 3.1% in perinatal complications.

**Table 2:** Maternal and neonatal outcomes pre- and post-SBT SBT, simulation-based training; NNT, number needed to treat; RR, risk ratio

Outcome (ref)	Pre-SBT (n, %)	Post-SBT (n, %)	p-value	RR (NNT)
Maternal transfusion (≥1 unit) [8-10]	645/20,067 (3.2%)	644/20,078 (3.2%)	0.99	
Neonatal Apgar <7 at 5 min [11-14]	5,760/72,246 (8.0%)	1,296/31,022 (4.2%)	<0.0001	0.52 (26)
Composite outcomes (Apgar <7 at 5 min and maternal transfusion)	6,405/92,313 (6.9%)	1,940/51,100 (3.8%)	<0.0001	0.55 (32)

Olson et al.’s study [15] provided valuable insights into the impact of SBT on shoulder dystocia management. This retrospective cohort study focused on standardizing obstetric maneuvers to improve neonatal outcomes and highlighted improvements in clinical team performance following simulation training. However, their reported outcomes, including neonatal Apgar scores, were presented as interquartile ranges (IQR) rather than proportions or means. This lack of compatible numerical data precluded integration of their findings into the pooled statistical analysis.

While maternal transfusion rates (3.2%) remained stable between groups, neonatal Apgar scores <7 at five minutes showed a significant reduction from 8.0% to 4.2%. These findings underscore the broad effectiveness of SBT in improving neonatal outcomes, with more variable effects observed for maternal outcomes.

Discussion

This systematic review highlights the significant impact of SBT on maternal and neonatal outcomes in delivery rooms. By analyzing diverse clinical scenarios, including PPH, neonatal asphyxia, and shoulder dystocia, the study provides robust evidence of SBT’s effectiveness in reducing adverse outcomes. These findings underline the value of SBT in improving clinical performance, enhancing team-based performance and collaborative clinical management, and improving outcomes in perinatal emergencies.

Strengths of the Study

A major strength of this review is its comprehensive evaluation of both maternal and neonatal outcomes across diverse clinical contexts. The inclusion of eight studies spanning over 177,000 deliveries provides a strong foundation for understanding SBT’s clinical relevance. Although maternal and neonatal outcomes are analyzed separately, resulting in some double-counting of deliveries, this dual focus reflects the interconnected nature of maternal and neonatal care and captures the holistic impact of simulation training.

Another strength is the inclusion of studies from a decade-long period (2014-2023), offering valuable insights into the evolution of simulation methodologies and their sustained clinical importance. This temporal scope allows for a broader understanding of how SBT has contributed to improved outcomes over time, even as simulation technologies and protocols have advanced. Moreover, the focus on high-resource environments ensures the applicability of findings to healthcare systems with established simulation programs and advanced clinical infrastructure.

Limitations of the Study

Despite its strengths, this review has several limitations. First, the heterogeneity of included studies, in terms of design (e.g., retrospective, prospective, and observational), training modalities (e.g., hybrid simulation, high-fidelity mannequins, and actor-based training), and outcome measures (e.g., transfusion rates rather than accurate quantification of maternal blood loss, Apgar scores, and dystocia-related trauma), limits the uniformity of conclusions. Second, the analysis includes both maternal and neonatal outcomes, which can result in some double-counting of deliveries when assessing pooled data. While this reflects the interconnected nature of obstetric care, it may slightly overestimate the overall population size.

Additionally, the included studies span a decade, during which simulation technologies and training methodologies have evolved substantially. Earlier studies may therefore not fully reflect the capabilities of contemporary high-fidelity simulators or standardized training protocols, introducing potential temporal bias, as illustrated by foundational work such as the shoulder dystocia training study by Draycott et al. published in 2008 [16]. Furthermore, the focus on high-resource settings limits the generalizability of these findings to low-resource environments, where logistical and financial constraints pose additional challenges for the implementation of simulation-based programs.

A broader limitation highlighted by this review is the absence of an international consensus on SBT standards, including training protocols, reported data, and outcome measures. Variability across programs, institutions, and countries complicates the evaluation and comparison of SBT effectiveness. In addition, the temporal span of the included studies reflects an evolution in simulation technologies and training practices, which may introduce temporal bias. The exclusive focus on high-resource settings further limits the generalizability of these findings, as SBT may yield larger observable effects in low- and middle-income countries due to higher baseline morbidity and mortality; however, differences in healthcare systems, resources, and outcome definitions restrict direct comparability. Finally, most included studies assess short-term outcomes, leaving long-term effects such as skill retention, sustained improvements in care, and cost-effectiveness largely unexamined.

Challenges for the Future

The increasing adoption of SBT poses several challenges for future research. With most healthcare professionals now trained using simulation, distinguishing between trained and untrained groups has become increasingly difficult, creating a saturation effect that calls for innovative study designs. A first priority will be to develop and validate standardized protocols, as the current absence of uniform training standards generates inconsistent outcomes and limits comparability across studies. Establishing internationally coordinated curricula applicable across diverse healthcare settings could provide a more coherent foundation for future international evaluation.

At the same time, future research should focus on extending SBT programs to low-resource settings, where maternal and neonatal mortality remain highest. Context-adapted initiatives, such as the Helping Babies Breathe program, suggest that low-cost, simulation-based approaches can improve essential neonatal resuscitation skills and early neonatal outcomes in low- and middle-income countries [17]. Adapting SBT to these environments may therefore require simplified training tools and scalable program designs. Beyond implementation, it will be essential to examine long-term outcomes, as most existing studies report only short-term effects. Longitudinal evaluations are needed to assess skill retention, changes in clinical decision-making, and sustained improvements in maternal and neonatal outcomes.

Methodological innovation will also play a key role. Future studies should make greater use of individual patient data and advanced analytical techniques, including subgroup analyses and meta-regression, to better capture the nuanced impact of SBT across diverse populations and contexts. Finally, as simulation programs require substantial investment, evaluating their cost-effectiveness, particularly in resource-limited settings, will be crucial to inform decisions on large-scale implementation and global dissemination.

## Conclusions

SBT has demonstrated a meaningful reduction in adverse maternal and neonatal outcomes across diverse clinical scenarios. While the heterogeneity of current practices highlights the need for standardized protocols, the evidence supports the integration of SBT as a core component of obstetric and neonatal education. Continued research is essential to assess long-term effectiveness, promote global standardization, and ensure broader implementation in varied healthcare settings.

## Appendices

Bibliographic research methodology

References were sourced from five databases, specifically PubMed (https://pubmed.ncbi.nlm.nih.gov), Cible + (https://cibleplus.ulb.ac.be), CISMeF (https://www.cismef.org), LiSSa (https://www.lissa.fr), and the Cochrane Library (https://www.cochranelibrary.com). The bibliographic search focused on relevant English- and French-language literature from 2014 onward. To ensure exhaustive data extraction and analysis, only articles available in full text were included. The research question and the resulting search equations were formulated using the PICOC acronym, detailed in Table 3.

**Table 3:** PICOC details

Patient	Pregnant women during labor and up to 28 days postpartum 0r newborns from 22 weeks of gestation and up to 28 days of life
Intervention	Initial or ongoing simulation-based training in delivery rooms
Comparison	No initial or ongoing simulation-based training
Outcome	Effects on maternal and infant morbidity and mortality
Context	Peri-partum in developed and emerging countries

The resulting research question is as follows: Does simulation-based training for health professionals in delivery rooms alter perinatal maternal and infant morbidity and mortality? The equations used are listed in Table 4.

**Table 4:** Search equations

Database	Search equation used	Search date
PubMed 1	(“Simulation training”[MeSH] OR “simulation based training”[MeSH]) AND “delivery rooms”[MeSH]	22/03/24
PubMed 2	“Simulation training”[MeSH] AND (“obstetrics”[MeSH] OR “gestation”[MeSH] OR “pregnancies”[MeSH])	22/03/24
PubMed 3	“Simulation training”[MeSH] AND (“perinatal mortality”[MeSH] OR “neonatology”[MeSH] OR “parturition”[MeSH])	22/03/24
Cible +	(“Simulation based learning” OR “simulation based training” OR “simulation training”) AND (“delivery room*” OR “labour room*”)	25/03/24
CISMeF	(“simulation” OR “simulation-based training”) AND (“delivery room” OR “birth” OR “obstetrics”)	01/04/24
LiSSa	(“simulation” OR “simulation-based training”) AND (“delivery room” OR “birth” OR “obstetrics”)	01/04/24
Cochrane	(“Simulation based learning” OR “simulation based training” OR “simulation training”) AND (“delivery room*” OR “labour room*”)	03/04/24

Articles were selected according to pre-established inclusion and exclusion criteria, which are listed in Table 5.

**Table 5:** Inclusion and exclusion criteria

Inclusion Criteria	Exclusion Criteria
Initial or ongoing simulation-based training. Newborns over 22 weeks of gestation and within the first 28 days of life. Pregnant women during labor and up to 28 days postpartum. Developed or emerging countries. Effects of simulation-based training on perinatal maternal and infant morbidity and mortality measured in real-life situations.	Simulation-based training in anesthesia techniques. No simulation-based training, either initial or ongoing. Methods for developing simulation-based training. Effects of simulation-based training in real-life situations not measured. Developing countries. Articles of the "review" type in the broad sense.

Articles obtained using the search equations were initially screened based on title and abstract to eliminate duplicates and those not relevant to the review topic. The full texts of the remaining studies were then analyzed to determine their potential inclusion in the review according to the pre-established selection criteria.

## References

[1] Chakravarthy B, Ter Haar E, Bhat SS, McCoy CE, Denmark TK, Lotfipour S (2011). Simulation in medical school education: review for emergency medicine. West J Emerg Med.

[2] Bienstock J, Heuer A (2022). A review on the evolution of simulation-based training to help build a safer future. Medicine (Baltimore).

[3] (2024). WHO. Neonatal and maternal mortality: fact sheets. World Health Organization. https://www.who.int/fr/news-room/fact-sheets/detail/newborn-mortality.

[4] Tosello B, Blanc J, Kelway C (2018). Medical simulation as a tool in the training of perinatal professionals (French). Gynecol Obstet Fertil Senol.

[5] Morris TP, White IR, Crowther MJ (2019). Using simulation studies to evaluate statistical methods. Stat Med.

[6] Norman G, Dore K, Grierson L (2012). The minimal relationship between simulation fidelity and transfer of learning. Med Educ.

[7] Hedges LV, Olkin I (2014). Statistical Methods for Meta-Analysis.

[8] Egenberg S, Øian P, Eggebø TM, Arsenovic MG, Bru LE (2017). Changes in self-efficacy, collective efficacy and patient outcome following interprofessional simulation training on postpartum haemorrhage. J Clin Nurs.

[9] Dillon SJ, Kleinmann W, Fomina Y (2021). Does simulation improve clinical performance in management of postpartum hemorrhage?. Am J Obstet Gynecol.

[10] Lutgendorf MA, Ennen CS, McGlynn A, Spalding CN, Deering S, Delorey DR, Greer JA (2024). Interprofessional obstetric simulation training improves postpartum haemorrhage management and decreases maternal morbidity: a before-and-after study. Int J Obstet Gynaecol.

[11] Xu C, Zhang Q, Xue Y, Chow CB, Dong C, Xie Q, Cheung PY (2023). Improved neonatal outcomes by multidisciplinary simulation-a contemporary practice in the demonstration area of China. Front Pediatr.

[12] Chitkara R, Bennett M, Bohnert J (2023). In situ simulation and clinical outcomes in infants born preterm. J Pediatr.

[13] Dahlberg J, Nelson M, Dahlgren MA, Blomberg M (2018). Ten years of simulation-based shoulder dystocia training- impact on obstetric outcome, clinical management, staff confidence, and the pedagogical practice - a time series study. BMC Pregnancy Childbirth.

[14] Fransen AF, van de Ven J, Schuit E, van Tetering A, Mol BW, Oei SG (2017). Simulation-based team training for multi-professional obstetric care teams to improve patient outcome: a multicentre, cluster randomised controlled trial. Int J Obstet Gynaecol.

[15] Olson DN, Logan L, Gibson KS (2021). Evaluation of multidisciplinary shoulder dystocia simulation training on knowledge, performance, and documentation. Am J Obstet Gynecol MFM.

[16] Draycott TJ, Crofts JF, Ash JP, Wilson LV, Yard E, Sibanda T, Whitelaw A (2008). Improving neonatal outcome through practical shoulder dystocia training. Obstet Gynecol.

[17] Mduma E, Ersdal H, Svensen E, Kidanto H, Auestad B, Perlman J (2015). Frequent brief on-site simulation training and reduction in 24-h neonatal mortality—an educational intervention study. Resuscitation.

